# The impact of electrode resistance on the biogalvanic characterisation technique

**DOI:** 10.1088/1361-6579/38/2/101

**Published:** 2016-12-23

**Authors:** J H Chandler, D A Head, M E Hubbard, A Neville, D G Jayne, P R Culmer

**Affiliations:** 1 School of Mechanical Engineering, University of Leeds, Leeds, UK; 2 School of Computing, University of Leeds, Leeds, UK; 3 School of Mathematical Sciences, University of Nottingham, Nottingham, UK; 4 Leeds Academic Surgical Unit, St. James’s University Hospital, Leeds, UK; P.R.Culmer@leeds.ac.uk

**Keywords:** biogalvanic, galvanic cell, numerical modelling, surgical sensing, cancer, tissue measurement

## Abstract

Measurement of a tissue-specific electrical resistance may offer a discriminatory metric for evaluation of tissue health during cancer surgery. With a move toward minimally-invasive procedures, applicable contact sensing modalities must be scalable, fast and robust. A passive resistance characterisation method utilising a biogalvanic cell as an intrinsic power source has been proposed as a potentially suitable solution. Previous work has evaluated this system with results showing effective discrimination of tissue type and damage (through electroporation). However, aspects of the biogalvanic cell have been found to influence the characterisation performance, and are not currently accounted for within the system model. In particular, the electrode and salt-bridge resistance are not independently determined, leading to over-predictions of tissue resistivity.

This paper describes a more comprehensive model and characterisation scheme, with electrode parameters and salt-bridge resistivity being evaluated independently. In a generalised form, the presented model illustrates how the relative resistive contributions from the electrodes and medium relate to the existing characterisation method efficacy. We also describe experiments with physiologically relevant salt solutions (1.71, 17.1, 154 mM), used for validation and comparison. The presented model shows improved performance over the current biogalvanic measurement technique at the median conductivity. Both the proposed and extant system models become unable to predict conductivity accurately at high conductivity due to the dominance of the electrodes. The characterisation techniques have also been applied to data collected on freshly excised human colon tissue (healthy and cancerous). The findings suggest that the resistance of the cell under the test conditions is electrode dominated, leading to erroneous tissue resistance determination. Measurement optimisation strategies and the surgical applicability of the biogalvanic technique are discussed in light of these findings.

## 1. Introduction

Advances in intraoperative imaging and measurement systems may facilitate a move toward personalised surgery, with associated benefits for the patient (Tufano and Kandil 2010, Tiernan *et al* 2012). However, there is currently no prominent solution to the problem of delivering relevant real-time intraoperative information to the surgeon during these procedures. A particular focus has been on the development of intraoperative imaging systems to aid in the delineation of anatomical features and localisation of cancerous targets (Taruttis and Ntziachristos 2012). Successful imaging typically requires the application of a contrasting agent. Within fluorescence imaging, indocyanine green (ICG) and 5-aminolevulinic acid (5-ALA) have ordinarily been used (Stummer *et al* 2006, Schaafsma *et al* 2011, Taruttis and Ntziachristos 2012, Filonenko *et al* 2014, Kondo *et al* 2014). A number of alternatives such as photoacoustic imaging are concurrently under investigation (De La Zerda *et al* 2008, Taruttis and Ntziachristos 2012, Mehrmohammadi *et al* 2013). To improve the efficacy of augmented surgical imaging, it is likely that cancer-specific functionalised contrast agents will be required (Schaafsma *et al* 2011). As a consequence, delay in quantifying the safety of these probes slows the progression to the clinic (Taruttis and Ntziachristos 2012, Mehrmohammadi *et al* 2013).

More localised measurements may offer alternatives to imaging and can arguably deliver a faster route to surgical integration. The technology focus within this area falls generally into measurement of mechanical (Săftoiu *et al* 2007) or electrical properties (Halter *et al* 2009). As an example of the latter, biogalvanic tissue characterisation has been proposed as a relatively simple means of determining tissue resistive properties (Golberg *et al* 2009, 2011). The technique discharges a biogalvanic cell, constructed with two different metal (zinc and copper) electrodes connected through a tissue medium, through a range of external resistive loads. The measured voltage drop from each load allows the cell to be characterised in terms of an internal resistance. Biogalvanic characterisation presents an alternative method for tissue assessment to more established techniques which are typically variants of bioimpedance spectroscopy (BIS). The key virtues of the galvanic method are its simplicity, both in implementation (minimal control circuitry is required in comparison to BIS) and interpretation of the resultant measures obtained. The single measure for the tissue obtained (its DC resistance) may be more convenient to correlate with clinical measures of tissue health in comparison to multi-parameter measures from BIS (components of the impedance spectra).

Application of the biogalvanic technique to tissues has demonstrated its potential as a sensing modality. Measurements on Sprague-Dawley rats showed significant differences with tissue type and damage induced through electroporation (Golberg *et al* 2009). In addition, tests extended to porcine tissues again showed tissue type specificity although significant variation in the cell voltage and a dependence on tissue strain was shown (Chandler *et al* 2014). Investigation in the underlying galvanic system has shown the electrode polarisation properties and current limitation mechanisms to be dominant within the characterisation process (Chandler *et al* 2015a). In addition, artefacts from the electrode-tissue interface have been shown to influence greatly the biogalvanic characterisation (Chandler *et al* 2015b). The simplicity and scalability of the technique make it potentially suitable for the challenging environment of surgery. However, current characterisation models are simplistic and do not account for the influence of the electrodes on the measured voltages. This paper reports a more comprehensive model of the galvanic system during characterisation. The presented spatially-extended finite element model has been used to examine how the operating conditions of the system are reflected in the characterisation process. In addition, the model has been implemented to reduce false tissue resistance readings and to allow for more accurate characterisation under the conditions expected with tissue measurements.

## 2. Materials and methods

Application of zinc and copper electrodes to tissue results in a galvanic cell being established. Under the condition of zero net current flow the potential difference of the electrodes is the open circuit voltage ($\text{OCV}$). Series connection of the galvanic cell to an external load results in current flow through the cell and an associated potential drop across the resistor. The existing method for biogalvanic characterisation determines tissue resistance ${{R}_{\text{INT}}}$ through application and fitting of the model expressed by

1 \begin{eqnarray*}\boldsymbol{V}=\frac{\text{OCV}}{\left(\boldsymbol{R}_{{\mathbf{EXT}}}+{{R}_{\text{INT}}}\right)}\boldsymbol{R}_{{\mathbf{EXT}}}\end{eqnarray*}

to the measured voltages ***V*** and corresponding external loads $\boldsymbol{R}_{{\mathbf{EXT}}}$ (Chandler *et al* 2014). This model assumes that the losses in potential are attributed only to the internal tissue resistance, itself assumed proportional to the volume strictly between the electrodes, and the external load. Additional considerations are required for integration of the resistive properties of the cell electrodes, and for lateral currents through the tissue.

Previously published results using the biogalvanic characterisation method (Chandler *et al* 2014, 2015a, 2015b) have used cell electrodes in axial alignment. This condition is most pertinent to a laparoscopic surgical grasping tool, where the electrodes would contact either side of the target tissue for assessment to be made. An axially aligned geometry was therefore used as the case study of our work including numerical modelling and physical measurement. For comparison to be made between the existing resistance method and our model (referred to from now on as the numerical model), tests were initially applied to salt solution (NaCl) analogues of known conductivity. Comparisons were subsequently extended to tissue resistance values determined from biogalvanic measurements made on healthy and diseased human colon tissue.

### 2.1. Computational biogalvanic model

The galvanic cell was modelled as a closed circuit consisting of the tissue, two electrodes in axial alignment, and a variable external resistance, all placed in series. Voltage–current relations for each component were derived independently (see section 2.1.1), and solved numerically using Brent’s method (Press 2007), under the constraints of a constant current *I*, and total voltage drop equal to the open current voltage ($\text{OCV}$).

#### 2.1.1. Geometry-dependent tissue resistance.

**Figure 1.**
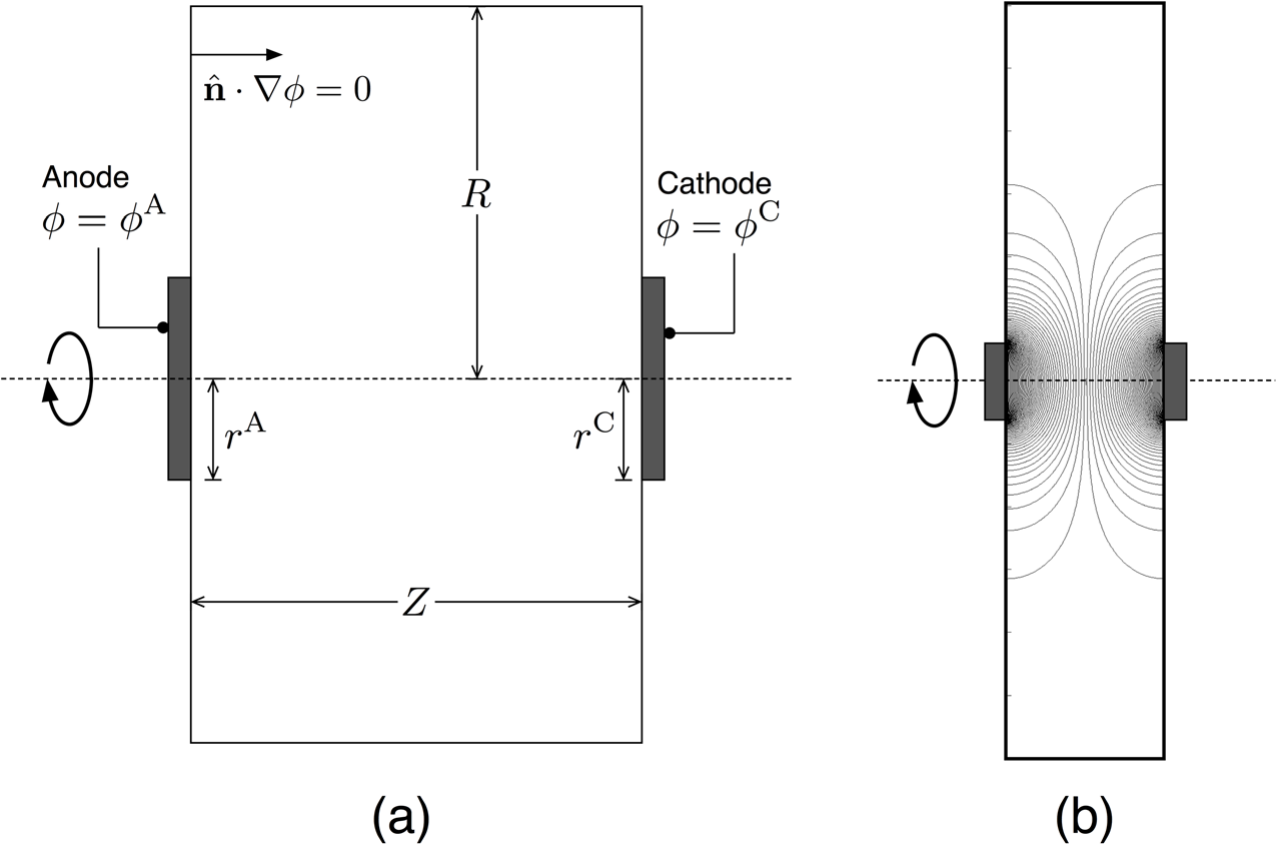
(a) Schematic of the model geometry for the medium (tissue) conductivity. The medium resides between the two electrodes held at fixed electric potentials, and there is zero current through all other boundaries. Cylindrical symmetry about the axes shown is assumed, so each electrode presents a circular surface. (b) Example of simulation output for a typical geometry (*R*  =  60 mm, *Z*  =  25 mm and *r*  =  6 mm). Contour lines correspond to 1% changes in $\phi $. Only one half-plane was actually solved for, the full solution was recovered by rotation about the axis.

The tissue was treated as a homogeneous conducting medium located between two opposing circular electrodes as shown in figure 1, where the local current density $\boldsymbol{i}\left(\boldsymbol{x}\right)$ is related to gradients in the electric potential $\phi \left(\boldsymbol{x}\right)$ via $\boldsymbol{i}=-\sigma \nabla \phi $ with $\sigma $ a uniform conductivity (Doig and Flewitt 1979, Munn and Devereux 1991). Steady state is assumed, and the lack of net electrical charge in the tissue means $\nabla \cdot \boldsymbol{i}=0$ and we recover the Laplace equation ${{\nabla}^{2}}\phi =0$. This is discretized using a linear Galerkin finite element scheme on a non-uniform triangular mesh with increased resolution near each electrode surface. This is solved iteratively using GMRES (Saad and Schultz 1986) within a Newton solve under the assumption of a cylindrically symmetric solution. A voltage difference $V$ was applied across the electrodes of radius $r$ and separation $Z$ (figure 1) and the net current $I$ measured, giving a linear, geometry-dependent *V*–*I* relation for the medium. The lateral spatial extent $R$ was increased until it had negligible effect (<2%) on the results. Results were validated by fit-free agreement with theoretical predictions for $Z\ll r$ and $Z\gg r$ (see supplementary information (stacks.iop.org/PM/38/101/mmedia)).

#### 2.1.2. Electrode and external resistance.

Each electrode was modelled by the non-linear Tafel equation for the overpotential $ \Delta V$ due to electrode resistance (Hamann *et al* 2007),

2 \begin{eqnarray*}{{i}^{A,C}}=i_{0}^{A,C}({{\text{e}}^{\Delta V/{{\alpha}^{A,C}}}}-{{\text{e}}^{- \Delta V/{{\beta}^{A,C}}}})\end{eqnarray*}

where the superscripts $A$, $C$ refer to anode and cathode parameters, respectively. The net current $I$ through each electrode was found by multiplying each ${{i}^{A,C}}$ by the corresponding electrode’s surface area. The external resistance was modelled as ohmic with known ${{R}_{\text{EXT}}}$.

#### 2.1.3. Fitting procedure.

The exchange current densities ${{i}_{0}}$ and Tafel voltages $\alpha $, $\beta $ for each electrode, along with the $\text{OCV}$ and the medium conductivity $~\sigma $, derive from the underlying electrochemistry (Hamann *et al* 2007), but since this is unknown they are treated as fit parameters. The tissue geometry parameters ${{r}^{A}}$, ${{r}^{C}}$, $R$ and $Z$ were treated as known and fixed. Fitting model predictions to data was performed by the Levenberg–Marquardt least-squares algorithm (Press 2007).

### 2.2. NaCl testing

Testing in aqueous sodium chloride (NaCl) was conducted to allow validation of the proposed model. Volumetric combination of analytical grade NaCl (99.9% purity, Fisher Scientific) and distilled water was performed to give solutions of 1.71, 17.1 and 154 mM NaCl (0.01, 0.1 and 0.9 wt% respectively). Cylindrical zinc and copper specimens of 12 mm diameter were fixed in non-conductive resin to leave only the circular end face of the metal exposed. Prior to testing, each sample was wet ground to 1200 grit and cleaned using distilled water.

#### 2.2.1. Polarisation scans.

Tests were initially performed to determine suitable ranges of Tafel parameters for application within the modelled system. Polarisation scans were performed using each electrode individually as the working electrode within a typical three-electrode cell. A combination Pt counter and Ag/AgCl reference electrode (thermo scientific) was used to complete the cell. All electrodes were submerged within a temperature-controlled salt solution for 30 min at open circuit prior to polarisation. Polarisation scans were performed for the established open circuit potential ($\text{OCP}$) to 300 mV in the anodic (increasing) direction for zinc electrode and the cathodic (decreasing) direction for copper. Five scans were performed for each metal using a scanning rate of 0.5 mV s^−1^.

#### 2.2.2. Biogalvanic characterisation.

Biogalvanic characterisation measurements were performed using a zinc and copper galvanic cell connected within 1.71, 17.1 and 154 mM NaCl solutions. Solutions were stirred and maintained at 25  ±  1 °C using a temperature-controlled magnetic stirring hotplate (MR Hei-Standard, Heidolph). Zinc and copper test electrodes were arranged axially within salt solution of appropriate concentration. Electrode spacing was adjusted to achieve a face separation of 25 mm, matching the configuration of the model system in figure 1. The external load on the cell was controlled through the use of a commercial potentiostat (CompactStat, Ivium Technologies). The external load was controlled in a reducing manner across 20 evenly log-spaced discrete loads ranging from 1 MΩ to 10 Ω. A switching rate of 0.1 Hz was employed for all tests, matching the configuration used in previous studies within NaCl (Chandler *et al* 2015a), and allowing sufficient settling time for transient voltages. Five characterisations were performed for each test concentration.

### 2.3. Tissue tests

Biogalvanic characterisation of human colon tissue containing a cancerous tumour (adenocarcinoma) was performed from a series of right hemicolectomy procedures. Freshly excised human colon tissue was obtained in accordance with NHS and Leeds University Teaching Hospital ethics procedures. Five repeat biogalvanic characterisation measurements were performed at the location of the tumour and within a healthy region (identified by the surgeon). Biogalvanic characterisations were performed using a commercial potentiostat (CompactStat, Ivium Technologies), with external loads controlled in a decreasing manner. External loads ranged from 110 kΩ to 4 Ω and were switched at a rate of 0.1 Hz for all tests.

## 3. Results

Parametric investigation of biogalvanic characterisation across known conductivity salt solutions and tissue measurements on diseased and healthy human colon tissue were successfully performed. Details of the results obtained are presented in the following sections.

### 3.1. Model parameter space

For clarity we consider symmetric systems where the anode and cathode have the same radius and electrochemical parameters, and the Tafel voltages $\alpha $ and $\beta $ are equal. Dimensional analysis then reduces parameter space to just two dimensionless variables, permitting systematic variation and easy visualization of results. We denote these two variables *a* and *b*, define them by

3 \begin{eqnarray*}a=\,\frac{Z}{r}\end{eqnarray*}

4 \begin{eqnarray*}b=\,\frac{\sigma \alpha}{r{{i}_{0}}}\end{eqnarray*}

and express all voltages and currents in units of $\alpha $ and $\pi {{r}^{2}}{{i}_{0}}$ respectively. *a* is a purely geometric quantity akin to an aspect ratio that is large when the electrode separation *Z* is much larger than their radius *r* (see figure 1(a)). The quantity *b* is a combination of geometric and biochemical parameters that has no *a priori* interpretation, but as shall be demonstrated below, controls the relative dominance of electrode over bulk conductivity in a manner that also depends on *a*.

**Figure 2.**
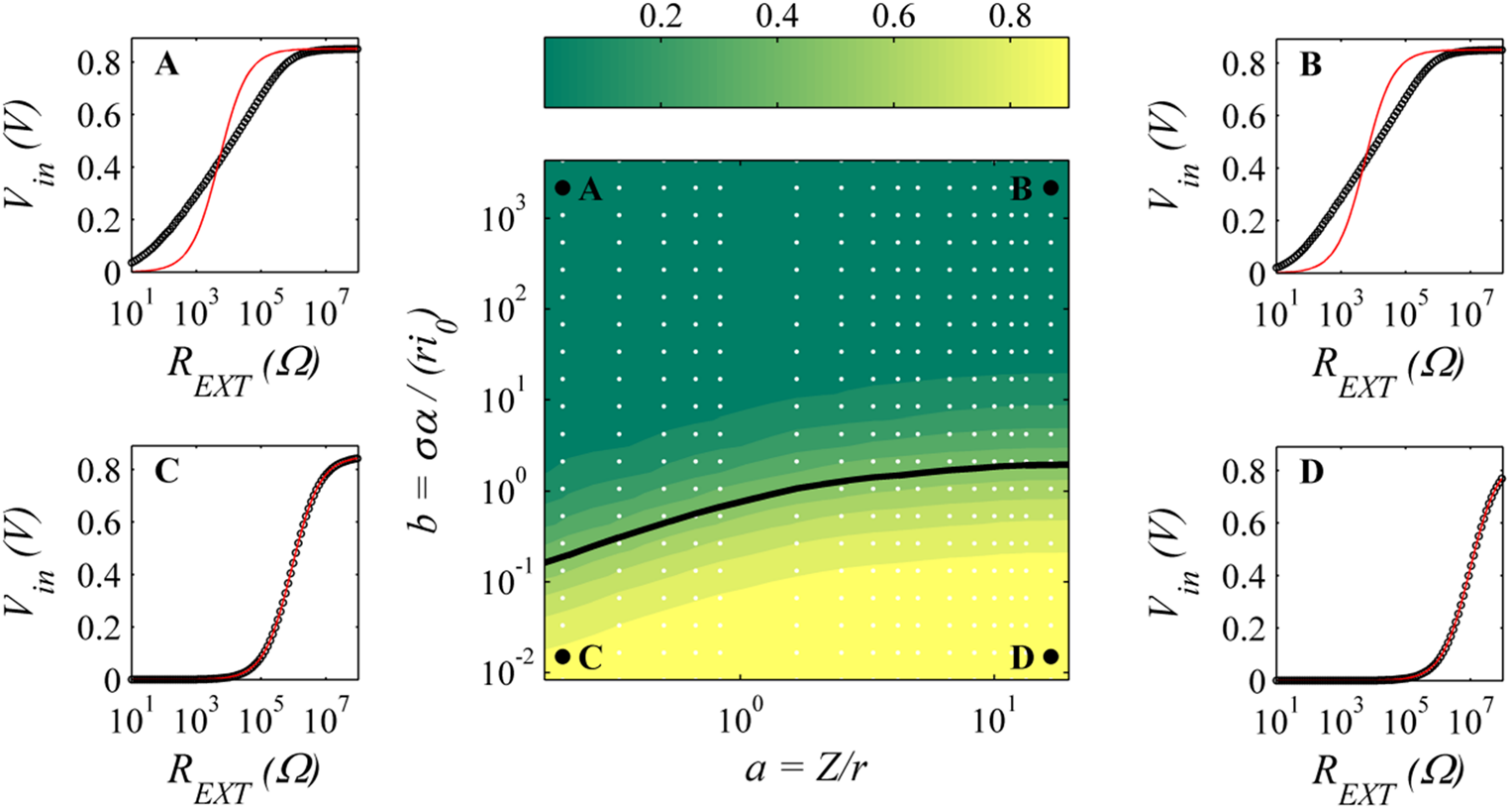
Contour plot of the fraction of voltage drop across the test medium for small voltages, versus $a$ and $b$. White points indicate actual data points, and the solid line shows the 50% value. Positions (A)–(D) show the numerical model output and the corresponding fit using the existing biogalvanic characterisation model for extreme locations within the parameter space.

Since the voltage drops across the electrodes and the medium are modelled separately, it is straightforward to calculate the fraction of the total voltage drop that is due to the medium alone. For large currents the medium always dominates, as the voltage drops across the electrodes are exponentially suppressed according to the Tafel expression, but for small currents both medium- and electrode-dominated regimes are influential, as plotted in figure 2. Fitting to an ohmic internal resistance is only appropriate for the medium-dominated regime (figures 2(C) and (D)); for large $b$, the exponential variation of the Tafel equation dominates and the fit becomes measurably poorer (figures 2(A) and (B)).

### 3.2. NaCl(aq) characterisation

**Figure 3.**
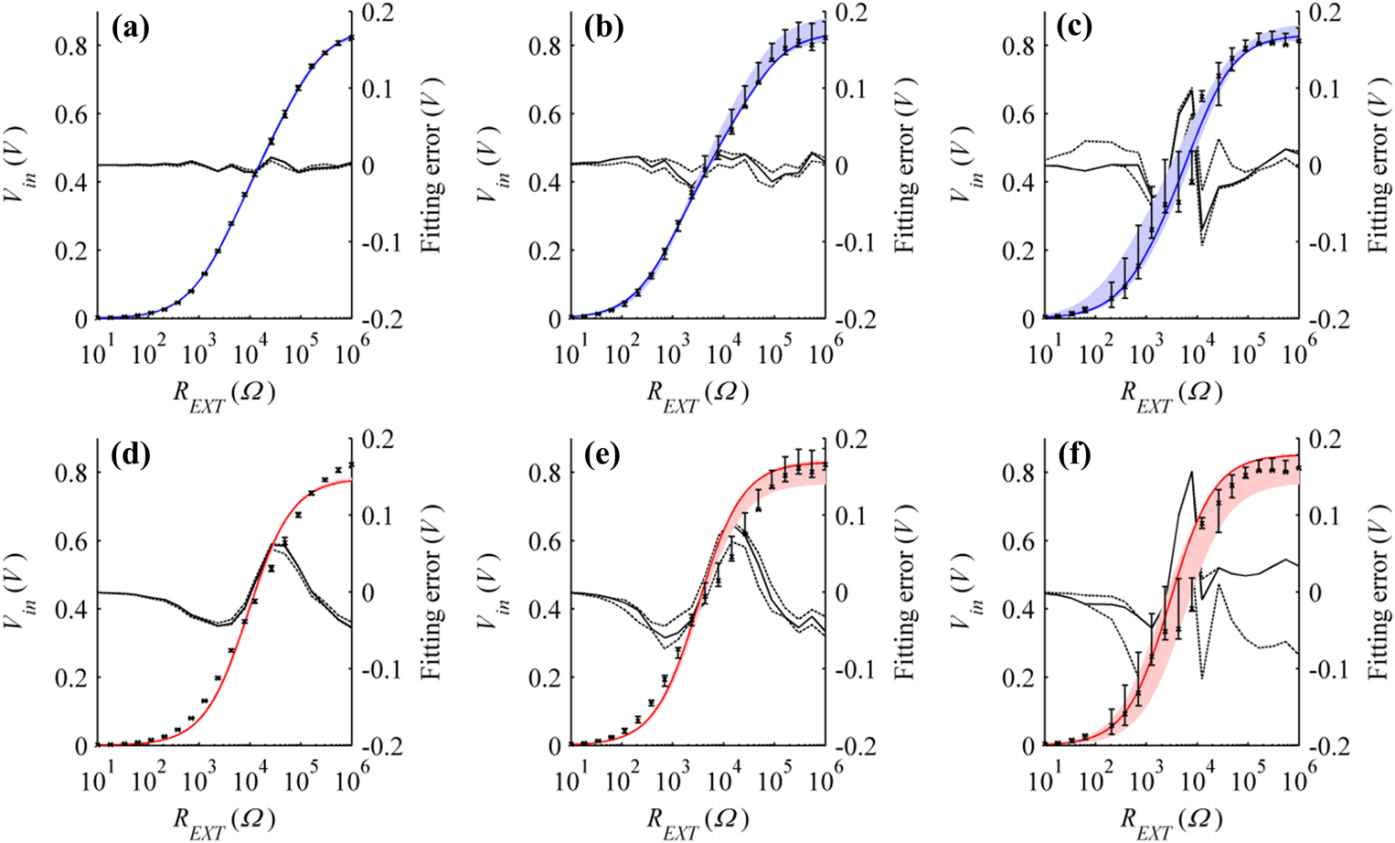
Median characterisation and range (shaded) of biogalvanic data measured in NaCl (*n*  =  5) using the numerical model for (a) 1.71 mM, (b) 17.1 mM, and (c) 154 mM, and using the existing biogalvanic fit for (d) 1.71 mM, (e) 17.1 mM, and (f) 154 mM; average fitting error (solid) and upper and lower fitting error ranges also shown (dashed).

Table 1 contains the full range of Tafel parameters measured within this study for the anodic and cathodic polarisation of zinc and copper respectively under varied salt solution concentration. Significant increases in parameter values can be seen for potential gradient and exchange current density for Cu as (NaCl) is increased. For the Zn electrode, the exchange current density also increases with (NaCl), while the potential gradient was found to reduce. The parameter ranges presented were used to inform fitting of the numerical model. Biogalvanic repeat measurements for salt solutions are shown in figure 3. A comparison of model fitting methods shows that the numerical model provides improved conformation to the measured data. The median fitting error along with the upper and lower range limits has also been presented for the two fitting methods across the range of external loads and (NaCl). For all concentrations, the numerical model shows reduced fitting error indicating improved agreement with the measured data. The average absolute fitting error across the range of external loads tested for the median fits of 1.71, 17.1 and 154 mM (NaCl) was 0.0036, 0.0096 and 0.024 V respectively for the numerical model, while the existing characterisation method produced respective average fitting errors of 0.024, 0.035 and 0.033 V for the same concentrations.

**Table 1.** Full range of measured Tafel parameters for zinc (anodic) and copper (cathodic) electrodes within varied (NaCl) (*n*  =  5).

	0.01		0.1		0.9	
	$\alpha $ (mV)	${{i}_{0}}$ (mA m^−2^)	$\alpha $ (mV)	${{i}_{0}}$ (mA m^−2^)	$\alpha $ (mV)	${{i}_{0}}$ (mA m^−2^)
Cu	48	0.5–2	100	2.5–4	82	4–8
Zn	44	3–5	32	4–16	19	25–40

**Figure 4.**
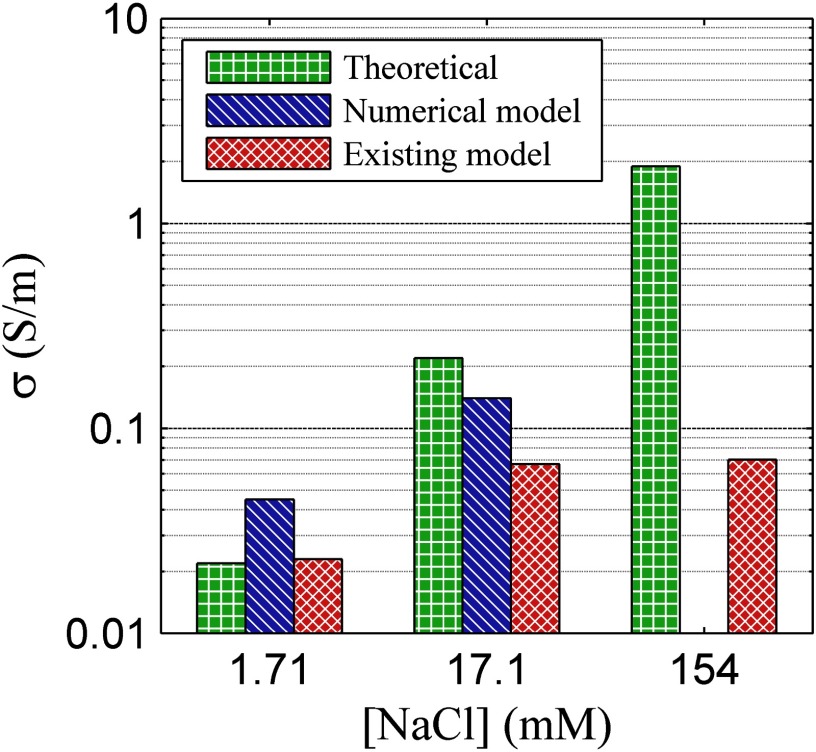
Comparison of theoretical conductivity for varied (NaCl) to the average determined conductivity using the numerical and existing characterisation models.

The fitting parameters for the numerical model have been included in table 2. A comparison of the conductivity fitting predicted across concentrations for the two methods is presented within table 3 (graphically presented within figure 4). Comparison of the model predictions with theory shows a dependence on the solution concentration (conductivity). For the low concentration of 1.71 mM (NaCl), the prediction of the conductivity is close to the theoretical value for both systems. Fitting using the simplistic model does show improved accuracy as the system is bulk-dominated and so electrode influence is minimised. For the middle concentration of 17.1 mM (NaCl) the fit conductivity using the existing method is much lower than theory, indicating an influence of the electrode resistance. The proposed method makes a more accurate prediction through the inclusion of both bulk and electrode contributions to the measured voltages. Under the high conductivity conditions of 154 mM (NaCl), the traditional fitting method becomes saturated and deviates strongly from the theoretical prediction. The proposed model explains this discrepancy as being due to an increasingly dominant contribution from the electrode surfaces, but is also unable to extract reliable estimates for the bulk conductivity due to its small contribution to the measured quantities (so no values are provided in tables 2 and 3).

**Table 2.** Numerical model fit parameters for repeat tests within varied (NaCl), showing variability as a function of concentration.

(NaCl) (mM)	$\sigma $ (S m^−1^)			$\alpha $ (mV)			${{i}_{0}}$ (mA m^−2^)			$\text{OCV}$ (V)		
	Mean	SD	% SD	Mean	SD	% SD	Mean	SD	% SD	Mean	SD	% SD
1.71	0.045	0.001	3.1	75	2	2.6	27	2	7.7	0.846	0.003	0.35
17.1	0.140	0.040	28.6	60	4	6.8	49	7	14.4	0.859	0.014	1.64
154	—	—	—	137	14	10.5	199	10	5.3	0.841	0.01	0.98

**Table 3.** Fit parameters for conductivity of varied (NaCl) using the current and proposed biogalvanic characterisation techniques; theoretical conductivities also given.

(NaCl) (mM)	Existing model			Numerical model			Theoretical
	Mean	SD	%	Mean	Abs error	%	—
1.71	0.023	0.0008	3.5	0.045	0.0014	3.1	0.022
17.1	0.0668	0.0040	6.0	0.14	0.04	28	0.22
154	0.0703	0.0381	54.2	—	—	—	1.9

### 3.3. Human colon characterisation

**Figure 5.**
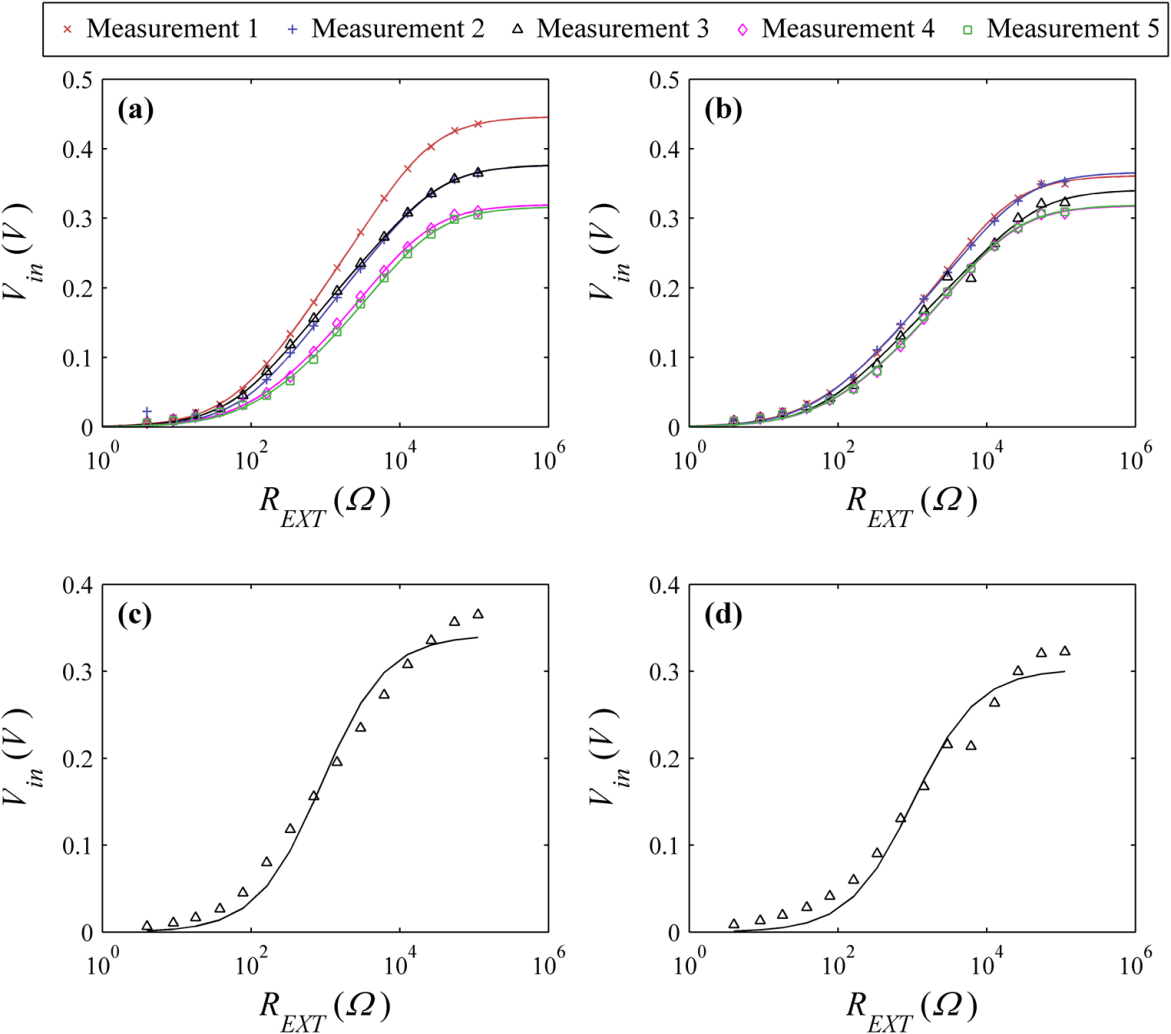
Repeat biogalvanic characterisation data from *ex vivo* human colon on (a) healthy region, and (b) cancerous region. Proposed model fits for each repeat are shown. Typical fits using the existing (bulk-dominant) model are also shown for the median healthy and cancerous biogalvanic measurements within (c) and (d) respectively.

Biogalvanic measurements on human colon show trends across the external resistor range of similar form to tests completed on NaCl solutions. Figures 5(a) and (b) indicates the measured data and illustrates the fitting solutions achieved for each repeat using the numerical model. For comparison, a bulk-dominant fit to the median repeat measured data for healthy and cancerous colon tissue using the existing model has been presented in figures 5(c) and (d) respectively. This model shows poor fitting to the measured data points. The fitting parameters achieved using the numerical model are presented in table 4. Due to the dominance of the electrodes under these test conditions, a suitable conductivity value was not achieved. Statistical assessment (Student’s *t*-test) of the two Tafel parameters (*α* and ${{i}_{0}}$) extracted independently showed no significant difference (*p*  >  0.05) between healthy and cancerous tissue.

**Table 4.** Model fit parameters for biogalvanic characterisation of *ex vivo* healthy and cancerous human rectal tissue.

Tissue type	$\sigma $ (S m^−1^)			$\alpha $ (mV)			${{i}_{0}}$ (mA m^−2^)			$\text{OCV}$ (V)		
	Mean	SD	%	Mean	SD	% SD	Mean	SD	% SD	Mean	SD	% SD
Healthy	—	—	—	45.5	5.1	11.2	140	35.1	25.1	0.37	0.02	6.3
Cancerous	—	—	—	40.1	1.7	4.2	111	24.0	21.6	0.34	0.01	2.9

### 3.4. Parameter space comparison

**Figure 6.**
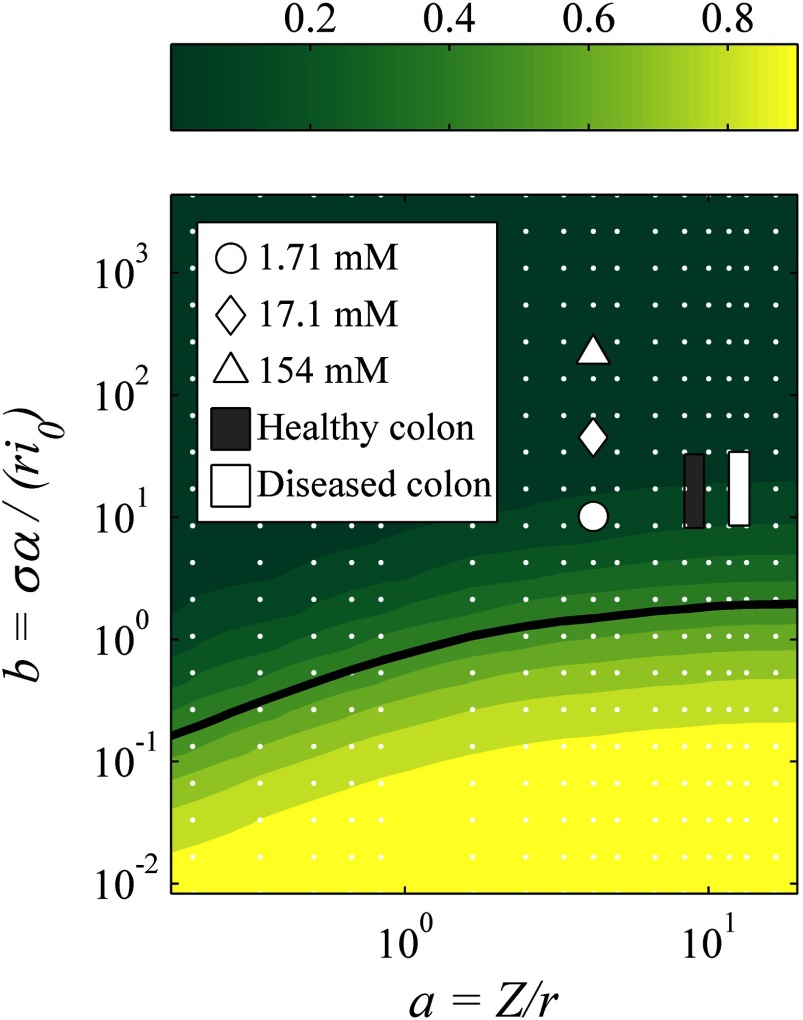
Model system parameter space including the defined positions for biogalvanic characterisation using the numerical model for varied (NaCl) and healthy and diseased colon tissues; conductivity values for colon tissue estimates from published soft tissue values (Faes *et al* 1999).

The fitting results from the numerical model-based characterisation of NaCl tests were used to define the parameter space positions for the varied concentrations. These estimated positions have been presented in figure 6. The lowest concentration tested is shown to be at the limit of the bulk-dominated regime, and higher concentrations extending well into electrode-dominated systems. As the numerical fitting to colon tissue data presented no reasonable conductivity value, representative values from published BIS tests were used for parameter space position estimation. Parameter space calculations were made using conductivity values ranging from 0.15 to 0.6 S m^−1^ (Faes *et al* 1999), which represent a wide range of soft tissue values, and the geometry and Tafel parameters from the presented tissue tests. The range of positions for healthy and cancerous tissues has been indicated on figure 6. The values clearly predict an electrode-dominant regime even at the highest tissue conductivity. This is consistent with the measured data and associated fitting presented in figure 5.

## 4. Discussion

Fitting to biogalvanic data using the existing model offers no means of separating the electrode resistance inherent within the two-electrode cell from the salt-bridge resistance of interest. The expanded numerical model reported in this paper has shown how knowledge and use of electrode properties can allow for independent assessment of these aspects under certain operating regimes. However, the physical constraints of the practical tissue sensing system present real problems for either technique.

### 4.1. Numerical model

The numerical model described has allowed for a level of parameterisation of the system, making assessment of the expected system behaviour over a wide range of conditions possible. Figure 2 shows the linear (small-current) response for a range of geometries and electrochemical properties quantified by the two dimensionless parameters *a* and *b* (equations (3) and (4) respectively). Reliable estimation of tissue conductivity *σ* is only possible far from the electrode-dominated regime $b\gg 1$. Currents far in excess of $\pi {{r}^{2}}{{i}_{0}}$ will also produce a medium-dominated response suitable for extraction of $\sigma $.

The spatially-extended finite element model for the medium resistance also allows the geometric dependence to be quantified. Two limiting cases can be readily understood. For $Z\ll r$, i.e. closely separated electrodes, the net medium resistance obeys

5 \begin{eqnarray*}R_{\text{med}}^{Z\ll r}=\frac{Z}{\pi {{r}^{2}}\sigma}\end{eqnarray*}

(see supplementary information) as postulated in Golberg *et al* (2009). However, for far-separated electrodes $Z\gg r$, each electrode can be represented as a point source or sink of electric current, and an alternative expression is derived as

6 \begin{eqnarray*}R_{\text{med}}^{Z\gg r}=\frac{2}{\pi r\sigma}.\end{eqnarray*}

Our experiments correspond to $a=Z/r\approx 4$, placing it intermediate between these two limits for which there is no analytical expression available, and numerical simulation is therefore required to determine the geometric dependence. There is no dependence on the medium geometry when the response is dominated by the electrode resistivity, as expected.

This suggests that application of the existing characterisation model, as presented within (Golberg *et al* 2009, Chandler *et al* 2014), is inappropriate for the determination of resistance and also conductivity through application of the geometric relationship of equation (5). The efficacy of the existing method has therefore been discussed for the salt solution analogues and human colon tissue tested, and contrasted to the findings from the proposed numerical model.

### 4.2. Influence of (NaCl) on the numerical model

Through application of measured Tafel parameters over the range of test solutions it has been demonstrated that the numerical model may also be utilised to characterise measured data and, under certain regimes, extract conductivity parameters. Figure 4 makes a comparison of the two methods, showing that for a very low salt solution concentration either system may offer a suitable prediction of the system conductivity. The simplistic bulk-dominated model in fact offers a more accurate prediction under these conditions, possibly due to the reduced number of fit parameters. At moderate concentration, values of conductivity expected within soft tissues (Faes *et al* 1999, Martinsen and Grimnes 2000), the influence of the electrodes becomes clear. The traditional characterisation method therefore greatly under-predicts the conductivity, and is indeed saturated to this minimum resistance due to the system electrodes. This minimum resistance is carried through to the highest tested concentration, making the value misrepresentative of the solution conductivity. Utilising the numerical model allows improved prediction at the intermediate concentration, due to the separation of electrode and bulk parameters. In addition, the numerical model predicts the onset of an electrode-dominated regime at high concentrations in which the predicted variation due to the bulk conductivity is within experimental errors, making reliable parameter estimation impossible. This null result nonetheless represents an additional benefit over the existing method, which instead continues to blindly generate increasingly unreliable estimates for the conductivity in this regime.

Figure 6 illustrates these systematic issues well, with NaCl concentrations under the geometric arrangement used being indicated within the $a$ versus $b$ parameter space. Increasing the concentration over two orders of magnitude shifts the system from partially electrode-dominated into strongly electrode-dominant. It is clear from both numerical and existing model characterisation of 154 mM (NaCl) that determination of meaningful conductivity at this position within the parameter space is not possible. However, only the numerical model indicates a null result under these conditions.

### 4.3. Application to human tissue

Figures 5(a) and (b), shows the data from a biogalvanic measurement on healthy and diseased human colon tissue with corresponding model fits using the numerical model. Figures 5(c) and (d), shows the median datasets for each tissue condition and the associated fit using the existing model. It is clear that the numerical method allows for better conformation to the measured data. The existing model fit does not match the data points across the external resistor range, and gives a best fit consistent with that of electrode dominance shown in figures 2(A) and (B).

Through combination of the average fitting parameters for $\alpha $ and ${{i}_{0}}$, along with published conductivity values for soft tissues (Faes *et al* 1999), a range of parameter space locations were estimated, as shown in figure 6. The positions indicated that healthy and cancerous tissues are both well into the electrode-dominant regime. This suggests that application of the existing model to biogalvanic tissue data collected under the tested geometries may not be appropriate for estimation of a tissue-specific resistance. Consideration of the biogalvanic resistance as a cell resistance (electrodes and tissue) rather than a tissue resistance may still allow for determination of a health-specific parameter. However, although the mean value for healthy colon (*M*  =  1200, SD  =  320 Ω) was higher than for cancerous colon (*M*  =  1066, SD  =  103 Ω), this difference, 133, was not significant *t*(8)  =  0.889, *p*  =  0.416. In addition, application of the existing model does not allow for analytic mitigation of the geometric influence on the system, and therefore conversion to conductivity or a geometrically normalised equivalent.

In contrast, the numerical model presented allows a geometrically independent conductivity to be determined. However, the electrode dominance within the tested tissue systems made the fitting process insensitive to this parameter, therefore giving a null result. The electrode potential gradient and exchange current density also did not show specificity for tissue type.

### 4.4. System adaptation

An alternative approach to avoid altering the underlying assumption of the existing model is to modify the cell properties and move the system into the bulk-dominant region. This can be achieved in three potential ways: (1) increasing the electrode surface area, (2) increasing the electrode separation, and (3) altering the electrode Tafel properties. The first two of these are simpler but have practical limitations when considering applications within surgery. As an example, to move the ‘$b$’ parameter toward a bulk dominated region (<2), electrodes of at least four times the radius would be required (24 mm). This geometry is practically unrealistic for the surgical setting, and is assuming the lowest tissue conductivity and does not account for the associated negative influence on the ‘$a$’ parameter. Electrode separation is not as easily evaluated for a practical case because the electrode-carrying device, the tissue geometry and region of interest, and the mechanical application of the electrodes all influence the separation at point of measurement. The assessed tissue cases reported represent a very large electrode separation, and it is therefore expected that measurements taken *in vivo* would in fact only be with reduced separation, thereby amplifying the electrode dominance of the system.

The final option of adjustment of Tafel parameters is not constrained by practical geometries, but by material properties. For a successfully passive system the galvanic cell should deliver a significant potential difference, therefore requiring specific half-cell reactions. For alternative materials that may offer more favourable Tafel parameters, the cell potential may be made too small for the measurement to be accurately taken. Ultimately, these parametric adjustments may reduce the influence of the electrodes upon the measurement system, however the required shift may not be practically achievable within the desired application. Under the existing configuration, the findings presented suggest that the biogalvanic method is not a suitable candidate for assessment of tissue resistance.

## 5. Conclusions

Application of a numerical model to the biogalvanic characterisation system has been detailed as a means of improving the understanding of the system. In addition, modelling in this way is the only means of extracting conductivity without including inappropriate assumptions regarding cell geometry. Successful development of a numerical model and system parameterisation has shown the apparent conflict between resistance of the electrodes and the tissue medium. To successfully mitigate resistive artefacts of the electrodes and be specific to the tissue medium, significant alteration of the cell geometry is required. However, such changes would render the sensing modality unsuitable as a surgical sensing technique. Therefore, the findings presented suggest that the biogalvanic method is not a suitable candidate for assessment of tissue resistance. Adaptation of the system to include additional potential measurement electrodes may offer means of improving efficacy and maintaining a practically suitable solution. Numerical modelling of this arrangement will be required to understand its inherent limitations and determine its suitability as a sensing modality.
